# Prevalent high-risk HPV infection and vaginal microbiota in Nigerian women

**DOI:** 10.1017/S0950268815000965

**Published:** 2015-06-11

**Authors:** E. O. DARENG, B. MA, A. O. FAMOOTO, S. N. AKAROLO-ANTHONY, R. A. OFFIONG, O. OLANIYAN, P. S. DAKUM, C. M. WHEELER, D. FADROSH, H. YANG, P. GAJER, R. M. BROTMAN, J. RAVEL, C. A. ADEBAMOWO

**Affiliations:** 1 Office of Strategic Information, Research and Training, Institute of Human Virology, Abuja, Nigeria; 2 Department of Primary Care and Public Health, University of Cambridge, UK; 3 Institute for Genome Sciences, University of Maryland School of Medicine, Baltimore, MD, USA; 4 Department of Microbiology and Immunology, University of Maryland School of Medicine, Baltimore, MD, USA; 5 Department of Nutrition, Harvard School of Public Health, Boston, MA, USA; 6 University of Abuja Teaching Hospital, Gwagwalada, Nigeria; 7 National Hospital, Abuja, Nigeria; 8 Department of Pathology, University of New Mexico Health Sciences Centre, Albuquerque, NM, USA; 9 Department of Epidemiology and Public Health, University of Maryland School of Medicine, Baltimore, MD, USA; 10 Institute of Human Virology and Greenebaum Cancer Centre, University of Maryland School of Medicine, Baltimore, MD, USA

**Keywords:** HIV/AIDS, human papilloma virus (HPV), public health

## Abstract

In this study, we evaluated the association between high-risk human papillomavirus (hrHPV) and the vaginal microbiome. Participants were recruited in Nigeria between April and August 2012. Vaginal bacterial composition was characterized by deep sequencing of barcoded 16S rRNA gene fragments (V4) on Illumina MiSeq and HPV was identified using the Roche Linear Array^®^ HPV genotyping test. We used exact logistic regression models to evaluate the association between community state types (CSTs) of vaginal microbiota and hrHPV infection, weighted UniFrac distances to compare the vaginal microbiota of individuals with prevalent hrHPV to those without prevalent hrHPV infection, and the Linear Discriminant Analysis effect size (LEfSe) algorithm to characterize bacteria associated with prevalent hrHPV infection. We observed four CSTs: CST IV-B with a low relative abundance of *Lactobacillus* spp. in 50% of participants; CST III (dominated by *L. iners*) in 39·2%; CST I (dominated by *L. crispatus*) in 7·9%; and CST VI (dominated by proteobacteria) in 2·9% of participants. LEfSe analysis suggested an association between prevalent hrHPV infection and a decreased abundance of *Lactobacillus* sp. with increased abundance of anaerobes particularly of the genera *Prevotella* and *Leptotrichia* in HIV-negative women (*P* < 0·05). These results are hypothesis generating and further studies are required.

## INTRODUCTION

Cervical cancer is the fourth most commonly diagnosed cancer and the fourth leading cause of cancer deaths in women worldwide, with an estimated 528 000 new cases and 266 000 deaths in 2012 [1]. It is the second most frequently diagnosed cancer in women living in Africa with 99 000 cases in 2012 and probably the commonest cancer in women living with HIV in Africa [2]. Cervical cancer incidence in developed countries has fallen by about 80% over the last two decades and may continue to decrease because of effective screening and human papillomavirus (HPV) vaccines. However, rates have largely remained stable in developing countries [3, 4].

Persistent infection of the cervix uteri by any of 15–18 HPV types classified by IARC as high-risk (hr) HPV types is the main risk factor for cervical cancer [5]. Some 5–15% of women are infected by HPV each year and this correlates with sexual debut, number of sexual partners and age [5, 6]. Approximately 10% of infected women fail to clear the virus leading to persistent infection [5–7]. In addition, even in women who seem to have cleared the infection, there is some evidence to suggest that there may be redetection of prior infections at a later age [8]. Factors that are associated with persistence of hrHPV remain largely unknown.

Although a causal role for hrHPV is well established in cervical carcinogenesis, relatively little is known about biological factors that influence the risk of persistence of hrHPV [9]. Disturbances in the vaginal microenvironment through vaginal douching practices [10], sexually transmitted infections [11, 12] and bacterial vaginosis (BV) [13] have been shown to be likely co-factors of persistence in HPV infection. While the mechanisms of these associations are not completely understood, the vaginal bacterial commensal communities (the microbiota) and the host adaptive/innate immune systems are probably involved.

The vaginal microbiota play a major role in preventing colonization and over-growth of microorganisms such as those associated with BV [14, 15] and urinary tract infections [16], as well as lowering the risk of acquisition and transmission of sexually transmitted infections, including HIV [17], Herpes simplex virus, *Neisseria gonorrhoea, Trichomonas vaginalis* and *Chlamydia trachomatis* [9, 18, 19]. Vaginal microbial dysbiosis, as in BV, could lead to increased susceptibility to hrHPV infection and reduced ability of the immune system to clear the infection [20, 21]. The primary mechanism by which the microbiota protects the female reproductive tract is hypothesized to be through production of lactic acid by *Lactobacillus* sp. via the anaerobic metabolisms of host-associated degradation products of glycogen stored in vaginal mucosal cells [22–24]. Other research groups have been investigating how vaginal lactobacilli have cytotoxic effects on cervical tumour cells using *in vitro* models [25].

Previous studies examining the link between the vaginal microbiota, hrHPV infection and its role in cervical carcinogenesis have typically used surrogates of vaginal microbiota status such as vaginal pH, Amsel or Nugent diagnosis of BV [26, 27] and cervical inflammation, or culture-dependent techniques [9, 13, 28]. Two recent studies utilized 16S rRNA gene analysis to evaluate the relationship between vaginal bacterial communities and HPV infection in a community of Korean and American women, respectively [29, 30]. Regarding the Korean women, the authors found that HPV-positive women had significantly higher microbial diversity with a lower proportion of *Lactobacillus* sp. than HPV-negative women. Additionally, they observed that HPV infection was strongly associated with Fusobacteria. In the study of 32 reproductive age women in the United States who were followed prospectively for 16 weeks, the authors found that community state type (CST) was associated with changes in HPV status and a low lactobacillus community with high proportions of the genera *Atopobium* had the slowest rate of HPV clearance. Another recent study in a Rwandan population utilized phylogenetic microarray methods and observed that women with microbiota dominated by *Lactobacillus crispatus* were less likely to have prevalent hrHPV infection compared to women with more diverse microbiota comprised of mixtures of anaerobes including species of the genera *Gadnerella, Atopobium* and *Prevotella* [31].

In this study, we evaluated the relationship between the composition of the vaginal microbiota and prevalent hrHPV infection in women attending a cervical cancer screening programme in Abuja, Nigeria.

## MATERIALS AND METHODS

### Data and sample collection

The study population for this analysis has been previously described [32, 33]. In summary, 278 women were recruited in Abuja, Nigeria between April and August 2012. Trained nurses administered questionnaires to collect information on socio-demographic characteristics and other risk factors. They conducted detailed gynaecological examinations and collected biological specimens. Mid-vaginal swabs and exfoliated ecto-cervical cells were collected from all participants using the Elution Swab system (Copan, Italy) and stored in 1 ml Amies' Transport media (Copan) using common clinical practices. The swabs were immediately frozen and stored at −80 °C until shipped to the University Of Maryland School Of Medicine, Institute for Genome Sciences where they were processed. Participants' HIV statuses were confirmed from their medical records. Study data were collected and managed using REDCap electronic data capture tools hosted at Institute of Human Virology, Nigeria [34].

### Vaginal microbiota characterization

#### Whole genomic DNA extraction from vaginal swabs

The swab suspensions were thawed on ice and vortexed vigorously to ensure an even distribution of cells. A total of 500 μl cell suspension was added to a 2 ml Fast Prep Lysing Matrix tube (Bio 101) containing 500 μl ice-cold PBS. Enzymatic lysis was initiated by adding 5 μl lysostaphin (4000 U/ml in 20 mm sodium acetate, pH 4·5), 13 μl mutanolysin (11·7 U/μl) and 3·2 μl lysozyme (1 mg/ml) and incubated at 37 °C for 37 min. Following this incubation, 10 μl Proteinase K (20 mg/ml), 50 μl of 10% SDS and 2 μl RNase A (10 mg/ml) were added. The mixture was incubated at 55 °C for 45 min. Samples were then subjected to mechanical lysis by bead beating in a Fast Prep 24 machine (MPBio, USA) at 6·0 m/s for 40 s. The lysates were centrifuged at 7000 rpm for 60 s to pellet the beads and filtered using the Zymo-Spin IV spin columns (Zymo Research, USA). DNA purification was performed on 500 μl lysate using the Qiagen Virus/Bacteria kit on a QIAsymphony SP instrument (Qiagen, USA) according to manufacturer's instructions. Total genomic DNA quality was checked by agarose gel electrophoresis (1% E-gel, Invitrogen, USA).

#### DNA amplification and sequencing of barcoded 16S rRNA gene fragments

Universal primers 515 F and 806R were used for PCR amplification of the V4 hypervariable region of the 16S rRNA gene according to the procedure of Caporaso *et al.* [35]. The 806R primer included a unique 12 bp sequence tag to barcode each sample. The V4 region of the 16S rRNA genes was amplified in 96-well microtitre plates using Phusion High-Fidelity DNA polymerase (Thermo Fisher, USA) and 50 ng template DNA in a total reaction volume of 50 μl. Reactions were run in a DNA Engine Tetrad 2 thermo cycler (Bio-Rad, USA) using the following cycling parameters: 30 s at 98 °C, followed by 25 cycles of 10 s at 98 °C, 15 s at 55 °C, and 15 s at 72 °C, with a final step of 10 min at 72 °C. Negative controls without template were included for each barcoded primer pair. Amplicons were confirmed by gel electrophoresis (2% E-gel) and quantified using the Quant-iT™ PicoGreen^®^ dsDNA assay (Life Technology, USA). Equimolar amounts (100 ng) of each of 278 PCR amplicons were mixed in a single tube. Primers and reaction buffer were removed from the pool of samples using the AMPure kit (Agencourt, USA). The purified amplicon mixture was sequenced on an Illumina MiSeq instrument (Illumina, USA) combined with 50% of control Phi-X control library using a 250 bp paired-end protocol recommended by the manufacturer.

#### Sequence analysis

The QIIME software package (qiime.org\) was used to perform quality control for the sequence reads using the following criteria: (1) having minimum and maximum length of 75% of original read length after filtering; (2) having an average quality score of q20 over a sliding window of 25 bp. If quality dropped below q20, the read was trimmed at the first base pair of the window and then reassessed for length; and (3) having a perfect match to a barcode sequence after autocorrection in QIIME. Sequences were binned by samples using the sample-specific barcode sequence. Paired reads were assembled using PANDAseq [36]. Using the UCLUST package high-quality reads were first de-replicated using 99% similarity and screened for potential chimeric sequences using UCHIME [37]. Chimeric sequences were removed prior to taxonomic assignments. Each processed 16S rRNA gene sequence was classified using the RDP Naive Bayesian Classifier [38] trained with the Greengene 16S rRNA database [39]. A *de novo* phylogeny was built on the filtered alignment using the RAxML method [40], based on which the weighted UniFrac distance metric [41] was calculated and principal coordinates analysis (PCoA) was used to perform principal coordinates reduction to assess the difference in overall microbial community composition [42].

#### CST assignments

Using the method described previously by Gajer *et al.*, the sample from each participant was assigned to a vaginal CST using hierarchical clustering based on the Jensen–Shannon distances between all pairs of community states and Ward linkage [43]. Heat maps of the relative abundance of the bacterial taxa found in each vaginal bacterial community was generated using the vegan package in R [44].

### Detection of HPV by genotyping

HPV DNA genotyping was performed using the Roche Linear Array^®^ HPV genotyping test (HPV LA; Roche Diagnostics, USA) using the manufacturer's recommendations as described previously [33, 45]. This genotyping test qualitatively detects 37 high- and low-risk HPV genotypes (13 hrHPV types: 16, 18, 31, 33, 35, 39, 45, 51, 52, 56, 58, 59, 68; and 24 low-risk HPV types: 6, 11, 26, 40, 42, 53, 54, 55, 61, 62, 64, 66, 67, 69, 70, 71, 72, 73, 81, 82, 83, 84, IS39, CP6108) [46].

### Statistical analysis

The association between specific bacterial taxa and hrHPV infection was evaluated using the Linear Discriminant Analysis (LDA) effect size (LEfSe) algorithm [47]. LEfSe performs two-stage comparisons, when the *P* value of the first comparison is <0·05, only then does it report LDA. The higher the LDA score for a taxon, the more significant the relationship is [47, 48]. LEfSe was adapted to quantitatively characterize the biomarker phylotypes that could explain the differences observed between two biological conditions [47]. The alpha value for the non-parametric factorial Kruskal–Wallis sum-rank test [49] was 0·05, the threshold on the logarithmic LDA model score for discriminative features was 2·0. Weighted UniFrac distances, which measure the distance between vaginal communities using phylogenetic information to reveal patterns of sample aggregation [41], were used to compare the vaginal microbiota of individuals with and without prevalent hrHPV infection. The taxonomic ranks of the identified taxa in LEfSe are generated using Ribosomal Dataset Project (RDP) taxonomy [50] that were further marked on a cladogram representing the taxonomic hierarchical structure from genera to phylum.

Student's *t* test and *χ*^2^ or Fisher's exact tests were used to assess associations between continuous/categorical variables and prevalent hrHPV infection status, respectively. In evaluating the relationship between vaginal bacterial community and hrHPV infection, we utilized different biostatistical models and bioinformatics tools. In model A, we examined the association between vaginal CSTs (as a predictor with four categories – CST I, CST III, CST IV-B and CST VI with CST IV-B being set as the reference category) and prevalent hrHPV infection using an exact logistic regression model, and in model B, we used exact logistic regression to examine the association between each CST and prevalent hrHPV infection by dichotomizing the CSTs into a CST of interest and all other CSTs combined. For example, in evaluating the effect of CST I, the CSTs were dichotomized into CST I and all other CSTs combined. This was repeated for CST III, CST IV-B and CST VI. Because there are numerous stratifications in CSTs, we used this binary approach in order to detect additional signals in the data. We also tested for interactions on HIV status and conducted stratified analysis of the association between hrHPV infection and vaginal microbiome by HIV infection status.

For the multivariable logistic regression model (model C), we included predictors that had *P* ⩽ 0·10 in univariate analyses or were known confounders. We set statistical significance at *P* ⩽ 0·05. All statistical analyses were performed using Stata statistical software v. 12 (StataCorp, USA).

### Ethics statement

The study was conducted according to the Nigerian National Code for Health Research Ethics and the Declaration of Helsinki. Ethical approval to conduct this study was obtained from National Health Research Ethics Committee of Nigeria (NHREC/01/01/2007-01/08/2014) and the University of Maryland IRB (HCR-HP- 00 051 495-2). Informed consent was obtained from all participants prior to enrolment in the study.

## RESULTS

A total of 278 women participated in the study, of which 66 (23·7%) tested positive for hrHPV. We were unable to ascertain the HIV status of 16 participants and these participants were excluded from all analysis in which the outcome variable was HIV status. The prevalence of hrHPV infection was significantly higher in HIV-positive (35·1%) compared to HIV-negative (10·8%) women (*P* < 0·001). Women with hrHPV infection were also more likely to have had ⩽12 years of education compared to hrHPV-negative women (*P* = 0·01) (Table 1). hrHPV-positive women were slightly younger with mean (s.d.) age of 34·2 (7·3) years compared to hrHPV-negative women [37·9 (7·8) years] (*P* = 0·001). Some 38·2% of the participants were aged ⩾40 years. Median number of live births, age at sexual debut, prevalence of smoking, contraceptive use, having used vaginal douching in the 3 months preceding study participation and frequency of vaginal intercourse were not statistically significantly different between hrHPV-positive and hrHPV-negative women.

**Table 1.** Characteristics of study participants, by hrHPV status

Characteristic	hrHPV+ (*N* = 66) *n* (%)	hrHPV– (*N* = 212) *n* (%)	*P* value*
HIV status†			
HIV–	12 (18·5)	99 (50·3)	<0·001
HIV+	53 (81·5)	98 (49·7)	
Age† (years)			
18–20	1 (1·5)	1 (0·5)	0·04
20–29	16 (24·3)	32 (15·3)	
30–39	33 (50·0)	87 (41·6)	
40–49	14 (21·2)	76 (36·4)	
50–59	1 (1·5)	12 (5·7)	
⩾60	1 (1·5)	1 (0·5)	
Vaginal bacterial community state type (CST)			
CST I (*L. crispatus)*	4 (6·1)	18 (8·5)	0·30
CST III (*L. iners)*	24 (36·3)	85 (40·1)	
CST IV-B (low *Lactobacillus)*	38 (57·6)	101 (47·6)	
CST VI (Proteobacteria)	0 (0·0)	8 (3·8)	
Education			
No formal education	4 (6·1)	3 (1·4)	0·01
Koranic school	0 (0·0)	1 (0·6)	
Vocational	0 (0·0)	2 (0·9)	
⩽12 years	34 (51·5)	76 (35·8)	
>12 years	28 (42·4)	130 (61·3)	
Smoking			
Ever smoked ⩾100 cigarette sticks	1 (1·5)	7 (3·3)	0·69
Never smoked ⩾100 cigarette sticks	65 (98·5)	205 (96·7)	
Contraceptive use >5 years‡			
Oral	0 (0·0)	4 (8·5)	0·42
Hormonal	1 (6·7)	11 (23·4)	
Intradermal	0 (0·0)	1 (2·1)	
Intrauterine	3 (20·0)	6 (12·8)	
Barrier (diaphragm, male and female condoms)	11 (73·3)	25 (53·2)	
Vaginal douching			
Within last 3 months	39 (59·1)	122 (57·5)	0·82
No vaginal douching	27 (40·9)	90 (42·5)	
Frequency of intercourse in past year†			
⩾once a month	48 (72·7)	147 (69·7)	0·64
<once a month	18 (27·3)	64 (30·3)	
Number of live births, median (IQR)	2 (3)	2 (3)	0·98
Age at enrolment, mean (s.d.)	34·2 (7·3)	37·9 (7·8)	0·001
Age (years) at sexual debut, mean (s.d.)	20·0 (4·1)	19·8 (4·5)	0·73

hrHPV, High-risk human papillomavirus; IQR, interquartile range.

Values given are *n* (%) unless stated otherwise.

* Fisher's exact test was used for age, vaginal bacterial community, education, smoking and contraceptive use, and Pearson's *χ*^2^ test was used for HIV status, vaginal douching within last 3 months and frequency of intercourse in the past year

† HIV status was not confirmed for 16 participants. Age variable was missing for three participants; frequency of intercourse in past year was missing for one participant.

‡ Sixty-two participants reported contraceptive use for >5 years.

### Characterization of the vaginal microbiota in study participants

**Fig. 1.**
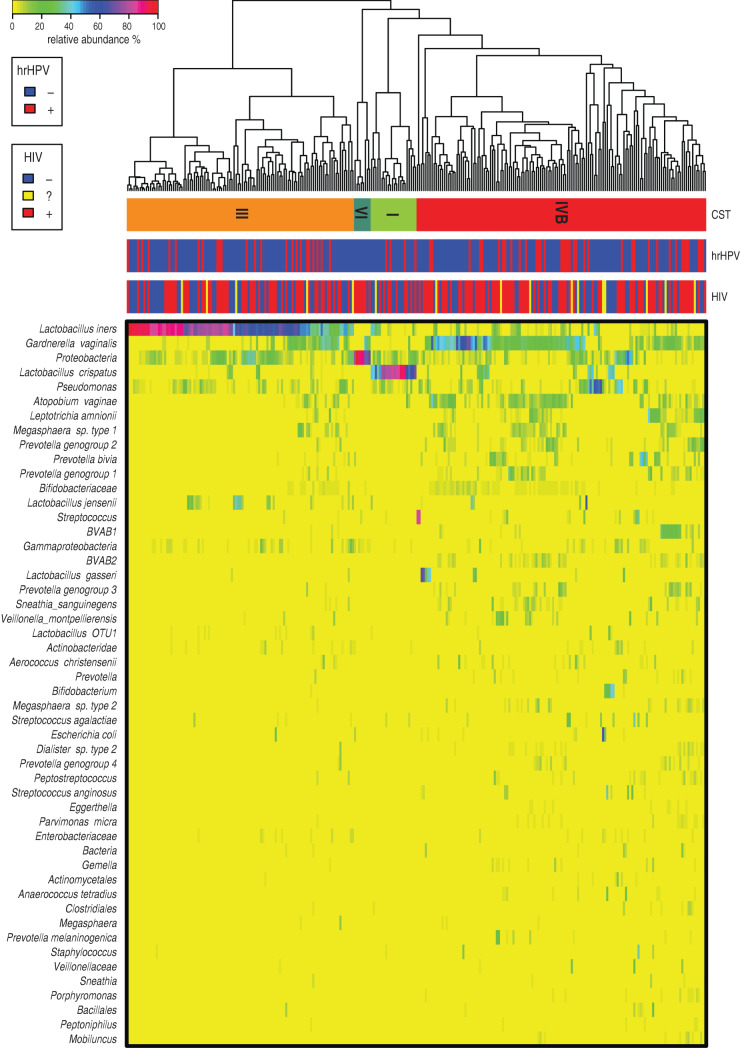
Heat map of relative abundance for the 50 most abundant bacterial taxa found in the vaginal bacterial communities of all participants in the study. Ward linkage clustering was used to cluster samples based on their Jensen–Shannon distance calculated in the vegan package in R [44]. Identified community state types (CSTs) are labelled as I, III, and IV, according to the previous naming convention [51]. hrHPV, High-risk human papillomavirus.

**Fig. 2.**
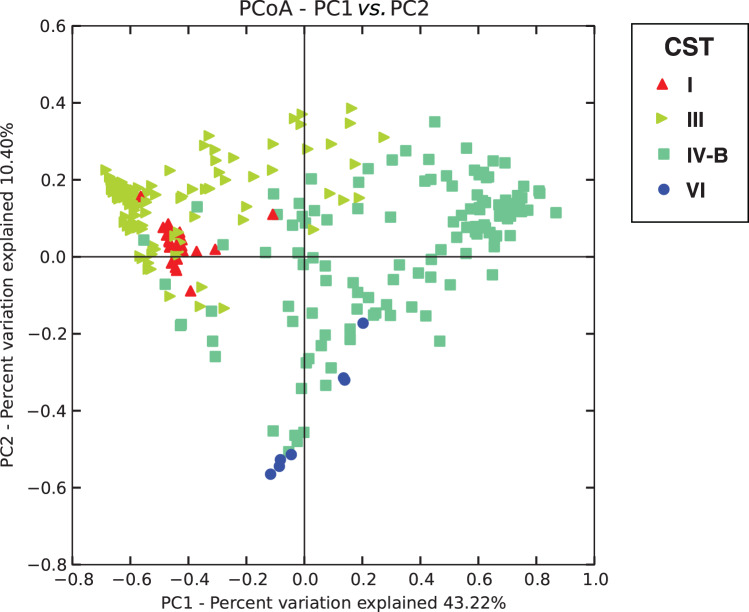
Weighted UniFrac principal coordinates analysis (PCoA) plot comparing sample distribution belonging to different community state types (CSTs). See Figure 1 for sample CST assignments used in this figure.

We identified four vaginal CSTs, three of which were previously characterized, and were labelled according to the described nomenclature [43, 51]. Figure 1 shows the bacterial taxa abundance in each of the 278 participants in the study by hrHPV and HIV status. The major vaginal CSTs identified were CST III dominated by *L. iners* and CST IV-B, which lacks significant numbers of lactobacillus and is composed of a diverse array of facultative and strictly anaerobic bacteria such as *Atopobium* and *Gardnerella* *vaginalis* among others. Some CSTs were less common, including CST I (dominated by *L. crispatus*) and CST VI, a novel CST dominated by members of the phylum proteobacteria described for the first time in this study. Communities dominated by *L. jensenii* or *L. gasseri* were not observed in this population, suggesting they were under-represented compared to a large study of North American women [51]. CST IV-B was the most prevalent CST detected, regardless of hrHPV and HIV status, with 139 (50%) participants having this type of vaginal microbiota. CST III was found in 109 (39·2%) participants, CST I in 22 (7·9%) participants and the proteobacteria-dominated CST VI in eight (2·9%) participants (Table 2). This distribution pattern was the same when participants were categorized by hrHPV status. A UniFrac analysis (Fig. 2) showed that all CSTs had distinct structures in terms of sample distance and distribution.

**Table 2.** Association between vaginal bacterial community state types (CSTs) and HIV, Abuja, Nigeria 2012

Vaginal bacterial CST	Total (*N* = 278) *n* (%)	HIV– (*N* = 111) *n* (%)	HIV+ (*N* = 151) *n* (%)	HIV? (*N* = 16) *n* (%)
CST I (*L. crispatus*)	22 (7·9)	12 (10·8)	9 (6·0)	1 (6·3)
CST III (*L. iners*)	109 (39·2)	50 (45·1)	54 (35·8)	5 (31·3)
CST IV-B (low *Lactobacillus* spp.)	139 (50·0)	48 (43·2)	81 (53·6)	10 (62·5)
CST VI (Proteobacteria)	8 (2·9)	1 (0·9)	7 (4·6)	0 (0)

	Fisher's exact *P* = 0·06*			
Univariate analysis*†	OR	(95% CI)	*P* value	
CST I (*L. crispatus*)	0·4	(0·2–1·3)	0·14	
CST III (*L. iners*)	0·6	(0·4–1·1)	0·12	
CST IV-B (low *Lactobacillus* spp.)	Ref.	(–)	–	
CST VI (Proteobacteria)	4·1	(0·5–190·8)	0·30	

OR, Odds ratio; CI, confidence interval.

* Sixteen participants with unconfirmed HIV status were excluded from these analysis.

† Exact logistic regression model.

The average abundances of the dominant bacteria in the communities were: *L. crispatus* in CST I (66·5%); *L. iners* in CST III (64·3%); *G. vaginalis* and *Atopobium vaginae* in CST IV-B (20·7% and 6·5%, respectively); and Proteobacteria in CST VI (77·5%) (Supplementary Table S1). CST assignments aside, *L. iners* and *L. crispatus* were detected in 97·8% and 69·4% of the participants, respectively.

Considering that CST VI has not been previously described in the literature, further evaluation of this community state was required; however, due to the limited number of participants with this CST, only exploratory analysis of this CST could be conducted at this time. From exploratory analysis, there appeared to be a trend towards an association between this CST and post-menopausal state (Fisher's exact *P* = 0·02) and most of the participants with this CST were HIV positive (Table 3).

**Table 3.** Characteristics of individuals in community state type (CST) VI compared to other CSTs

Characteristic	CST VI (*N* = 8) *n* (%)	Non-CST VI (*N* = 270) *n* (%)	*P* value*†
Nature of residence†			
Urban	6 (75·0)	99 (36·8)	0·12
Semi-urban	1 (12·5)	104 (38·7)	
Rural	1 (12·5)	66 (24·5)	
HIV status‡			
Negative	1 (12·5)	110 (40·7)	0·24
Positive	7 (87·5)	144 (53·3)	
Unknown	0 (0·0)	16 (5·9)	
Marital status			
Married	3 (37·5)	167 (62·1)	0·06
Single	0 (0·0)	40 (14·9)	
Separated	1 (12·5)	12 (4·4)	
Divorced	2 (25·0)	10 (3·7)	
Widowed	2 (25·0)	35 (13·0)	
Cohabiting	0 (0·0)	1 (0·4)	
Others	0 (0·0)	4 (1·5)	
Vaginal douching†			
Ever douched	5 (62·5)	170 (63·2)	1·00
Never douched	3 (37·5)	99 (36·8)	
Menopausal status			
Pre-menopausal	4 (50·0)	229 (85·1)	**0·02**
Post-menopausal	4 (50·0)	40 (14·9))	
Use of sanitary protection			
Panty liners	0 (0·0)	2 (0·7)	1·00
	8 (100·0)	268 (99·3)	
Tissue	1 (12·5)	37 (13·7)	1·00
	7 (87·5)	233 (86·3)	
Sanitary pads	6 (75·0)	219 (81·1)	0·65
	2 (25·0)	51 (18·9)	
Cloth napkins	2 (25·0)	28 (10·4)	0·21
	6 (75·0)	242 (89·6)	
Tampons	0 (0·0)	4 (1·5)	1·00
	8 (100·0)	266 (98·5)	
Age, years, mean (s.d.)	45·3 (6·7)	36·7 (7·7)	**0·002**
Number of sexual partners in the past year, median (IQR)	1 (1)	1 (0)	0·40

IQR, Interquartile range.

Values given are *n* (%) unless stated otherwise.

* Fisher's exact test.

† One participant did not have relevant data.

‡ HIV status not confirmed for 16 participants.

### Vaginal bacterial community and HIV infection

Because HIV has previously been shown to be associated with disruptions in the vaginal microbiota, which could be explained by suppressed immune system associated with the disease, and may remain even in HIV-positive persons on combination anti-retroviral therapy [52, 53], we evaluated the association between vaginal CST and HIV status in our study. There was a trend (*P* = 0·06) towards reduced HIV infection in women with a *Lactobacillus*-dominated microbiota compared to women with a *Lactobacillus*-deficient state (CST IV-B), [CST I: odds ratio (OR) 0·4, 95% confidence interval (CI) 0·2–1·3; CST III: OR 0·6, 95% CI 0·4–1·1] (Table 2).

### Vaginal bacterial community and prevalent hrHPV infection

In model A, CST I, III and VI were inversely associated with hrHPV infection compared to CST IV-B although these were not statistically significant (Table 4*a*). Further adjustments for age, sexual debut age, total number of sex partners in the previous year, visual inspection with acetic acid results, HIV status and reported use of male condoms in multivariable logistic regression model (model C) did not change the relationship (Table 4*a*).

**Table 4a.** Association between vaginal bacterial community types and hrHPV, Abuja, Nigeria 2012

	Model A*		Model B Univariate logistic model		Model C Multiple logistic regression model	
Total sample (*N* = 278)	OR (95% CI)	*P*	OR (95% CI)	*P*	OR (95% CI)	*P*
CST IV-B (low *Lactobacillus* spp.)	Ref.	–	1·5 (0·8–2·7)	0·20	Ref.	–
CST I (*L. crispatus*)	0·6 (0·1–2·0)	0·53	0·7 (0·2–2·2)	0·53	0·4 (0·1–1·7)	0·29
CST III (*L. iners*)	0·8 (0·4–1·4)	0·42	0·9 (0·5–1·6)	0·69	0·7 (0·3–1·3)	0·22
CST VI (Proteobacteria)	0·2 (0·0–1·7)	0·17	0·3 (0·0–1·9)	0·22	–	
Age, years			0·9 (0·9–1·0)	0·001	0·9 (0·9–1·0)	<0·05
Sexual debut age			1·0 (0·9–1·1)	0·73	1·2 (0·6–2·2)	0·28
Number of sexual partners in past year			1·1 (0·6–2·0)	0·64	1·3 (0·7–2·4)	0·43
VIA results (ref. – VIA positive)			0·4 (0·1–1·5)	0·19	0·4 (0·1– 1·7)	0·23
HIV Status (ref. – HIV negative)			4·7 (2·4–9·2)	<0·001	5·2 (2·4–11·0)	<0·05
Use of male condoms (ref. – Never use)			2·4 (1·3–4·2)	0·003	1·8 (0·9–3·5)	0·10

OR, Odds ratio; CI, confidence interval; VIA, visual inspection with acetic acid.

* Exact logistic regression model.

**Table 4b.** Association between vaginal bacterial community state type (CST) and any human papillomavirus (HPV) infection

Vaginal bacterial CST	Any HPV		Total* *n* (%)
	Negative *n* (%)	Positive *n* (%)	
CST I (*L. crispatus*)	18 (10·8)	4 (3·8)	22 (8·1)
CST III (*L. iners*)	66 (39·5)	40 (38·1)	106 (39·0)
CST IV-B (low *Lactobacillus* spp.)	76 (45·5)	60 (57·1)	136 (50·0)
CST VI (Proteobacteria)	7 (4·2)	1 (1·0)	8 (2·9)
Total	167 (100·0)	105 (100·0)	272 (100·0)
	Fisher's exact *P* = 0·047		

* Six participants were missing information on low-risk HPV infection and were excluded from this analysis.

In model B, we observed inverse associations for CST I, CST III and CST VI although none of the associations were significant, similar to what we saw in model A. Women with CST IV-B had a positive association with being hrHPV positive (OR 1·50) but this was also not statistically significant.

We also evaluated the relationship between any HPV infection (34 types detected) and vaginal bacterial community type. We observed that CST was associated with HPV detection (*P* = 0·047, Table 4*b*). There was an increased proportion of women with CST IV-B in those who had any HPV infection compared to women without any HPV infection.

Considering the potential for effect modification by HIV infection, we generated interaction terms for vaginal bacterial communities and HIV and tested these using the likelihood ratio test; these were also not statistically significant (*P* = 0·59).

**Fig. 3.**
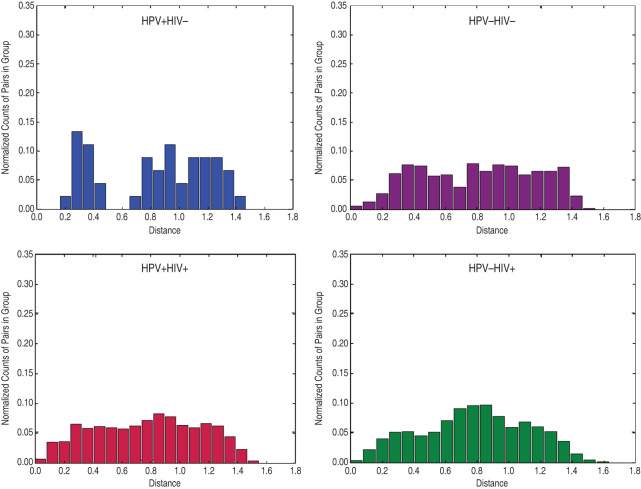
Histogram of weighted UniFrac distance between samples by human papillomavirus (HPV)/HIV metadata. The distribution of distance between samples of HPV + /HIV–, HPV–/HIV–, HPV + /HIV + , and HPV–/HIV+ women is shown.

**Fig. 4.**
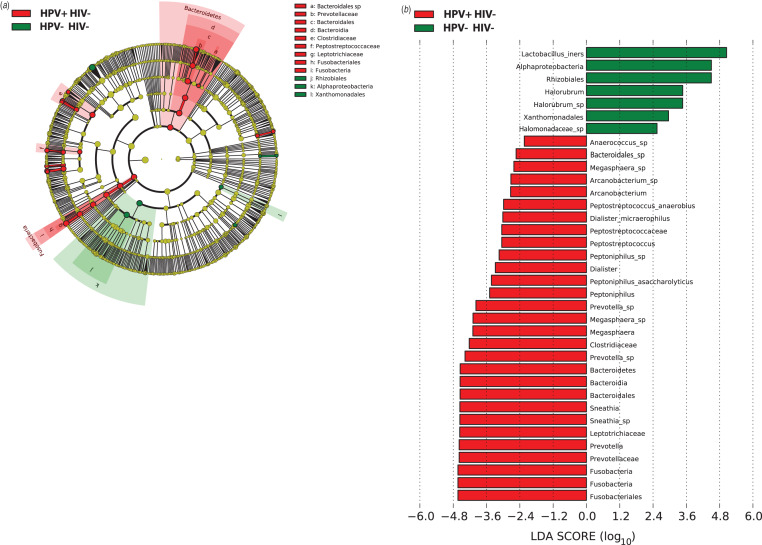
(*a*) Cladogram representing the taxonomic hierarchical structure of the identified phylotype biomarkers, generated using LEfSe [47]. Phylotype biomarkers are identified comparing samples collected from HIV–/HPV– and HIV–/HPV+ participants. Each filled circle represents one biomarker. Red, phylotypes statistically overrepresented under the condition of HPV + /HIV–; green, phylotypes overrepresented under the condition of HPV–/HIV–; yellow, phylotypes for which relative abundance is not significantly different between the two conditions. The diameter of each circle is proportional to the phylotype's effect size, phylum and class are indicated in their names on the cladogram and the order, family, or genera are given in the key. (*b*) Identified phylotype biomarkers ranked by effect size in HIV– women. The phylotype biomarkers are identified as being significantly abundant comparing samples collected from HPV– and HPV+ women with an alpha value <0·05. The graph was generated using the LEfSe program. The phylotypes are ranked according to their effect size that are associated with different conditions with the highest median. The Linear Discriminant Analysis (LDA) score [47] at the log_10_ scale is indicated at the bottom. The greater the LDA score is, the more significant the phylotype biomarker is in the comparison.

We then compared the vaginal microbiota from hrHPV-infected and -uninfected women using weighted UniFrac distances and found that the vaginal microbiota of hrHPV-positive women was significantly different from that of hrHPV-negative women but only in HIV-negative women (*P* = 0·02) and not in HIV-positive women (Fig. 3). Of HIV-positive women, there was no difference in the sample distance distribution of hrHPV-positive compared to hrHPV-negative women (*P* = 0·64). We used LEfSe to identify the specific bacterial taxa that were differentially present or abundant in the vaginal microbiota of HIV-negative women with and without hrHPV infection (Fig. 4). We focused on HIV-negative women because of the results of the weighted UniFrac distances that showed that the vaginal microbiota exhibited little variation in the specific bacterial taxa of HIV-positive women by hrHPV status (Fig. 3). hrHPV infection was strongly associated with abundance of various vaginal bacterial taxa, particularly members of the families Leptotrichiaceae and Prevotellaceae, and also Clostridiaceae and Peptostreptococcaceae. The most abundant genera in the hrHPV-positive group were *Prevotella* and *Leptotrichia* from the phyla Fusobacteria and Bacteriodetes, respectively. The occurrence of hrHPV infection was also strongly associated with lower abundance of *L. iners* with an LDA score of 5·05 (Fig. 4*b*)

## DISCUSSION

In this study, we examined the association between vaginal microbiota and prevalent hrHPV infection in Nigerian women. We found a moderate association between prevalent hrHPV and a low relative abundance of *Lactobacillus* sp. In subset analysis, LEfSe analysis and comparisons of the weighted UniFrac distances showed that this relationship was statistically significant in HIV-negative individuals. The most abundant genera in the HIV-negative hrHPV-positive participants in this study were *Prevotella* and *Leptotrichia. Prevotella* has been shown to be present in certain dysbiotic states such as occurs in BV [15]. Although *Leptotrichia* which belongs to the same family as the genera *Fusobacterium* and *Sneathia*, is considered to be part of the normal oral cavity and the female genital tract microflora, it has been isolated from infections of the female genital tract such as salpingitis and BV [54, 55]). Our result is similar to that obtained in a Korean study where HPV positivity was associated with a lower prevalence of *Lactobacillus* spp. and an increased abundance of anaerobic bacteria including *Prevotella, Sneathia, Dialister*, and *Bacillus* species [29].

Another key finding from our study was that CST IV-B, a state characterized by a substantial lack of *Lactobacillus* spp. and high levels of *G. vaginalis* and other facultative and strict anaerobes, was the most prevalent CST regardless of hrHPV and HIV status. This CST has previously been reported as the most common CST in African American women [51, 56]. Another study of Tanzanian women also found *G. vaginalis* and *L. iners* were the most prevalent members of the microbiota in their study population [57].

Further, despite CST assignments, *L. iners* and *L. crispatus* were detected in most samples from our study, with *L. iners* present in higher abundance in a larger number of samples. The presence of some lactic acid-producing bacteria in the majority of the communities suggests the capability for each community state to provide some measure of protection.

There were very few women with CST VI in this study, and additional characterization of this group of women revealed that they were mainly post-menopausal. The relative lack of *Lactobacillus* spp. in these women may be an indication of the changes in the composition of the vaginal microbiota as oestrogen levels decline [58]. These observations are consistent with the findings of a positive association between post-menopausal state and a low *Lactobacillus* spp. vaginal microbial state in a prospective cohort study of 87 women participating in a study assessing HPV in the perimenopause [59] and the findings of an increased abundance of proteobacteria in a twin cohort in Korea [29]. Further studies with larger sample sizes are required to fully characterize the changes in vaginal microbiota at menopause.

The determinants of vaginal microbiota composition in any population are not well known. The observed variation by ethnic groups described by Ravel *et al.* [51] may reflect a role for genetic factors but does not rule out non-genetic risk factors such as socio-cultural and health practices. The preponderance of *Lactobacillus*-depleted communities in Nigerian women may underlie well-known differences in the susceptibility of black women to BV and sexually transmitted infections [57].

With regard to HIV infection, our results showed a trend towards an association between the type of vaginal microbiota present and HIV, with *L. crispatus*-dominated CST I and *L. iners*-dominated CST III being inversely associated compared to the more diverse CST IV-B. Additionally, CST VI appeared to be more prevalent in HIV-positive participants. These observations are similar to the findings of Spear *et al.* in a subset of the participants in the Women's Interagency HIV Study [60], where it was observed that HIV-negative women had a higher abundance of *L. iners*. In another analysis by Spear *et al*., it was observed that there was a higher microbial diversity in women with HIV infection [52], and this is congruent with our findings. Our findings are also in agreement with the findings of Borgdorff *et al.* in a study of 174 female sex workers in Rwanda, where HIV prevalence was higher in women with vaginal microbiome clusters not dominated by *Lactobacillus* sp. [31]. Given the role of HIV in immune suppression, it is possible that the reduced immunity may account for the greater microbial diversity, or by contrast, women lacking *Lactobacillus* spp. were at greater risk for acquiring or transmitting HIV.

Our study is limited by its cross-sectional design, therefore we could only evaluate associations and are unable to infer causality. Another limitation of this study is that we evaluated associations between CSTs and prevalent hrHPV infection. Prevalent hrHPV has a weaker association with cervical cancer and pre-cancer compared to persistent hrHPV infection and HPV status is known to fluctuate in studies where samples are frequently collected [61]. However, we continued to follow-up these participants to ascertain whether these associations would change with persistent hrHPV infection. Our participants were recruited from cervical cancer screening clinics and may not represent women in the community in general. Some of the data used in this study were self-reported and thus subject to recall bias. Other limitations of self-reported data particularly those related to sexual practices include the tendency to provide only socially desirable responses.

To our knowledge this is the first study to evaluate the association between vaginal microbiota composition and structure, and hrHPV in African populations using culture-independent techniques to characterize the vaginal microbiota and hrHPV. Other studies have used culture-independent techniques to characterize the vaginal microbiota in HIV-positive women in African populations [31, 62, 63], but did not evaluate its relationship with hrHPV specifically. We found that vaginal microbial composition in African women is similar to that of African American women and that hrHPV infection was strongly associated with the abundance of various vaginal bacterial taxa, particularly *Prevotella* and *Leptotrichia.* These findings are hypothesis generating and warrant further investigations in larger cohorts followed longitudinally.

## SUPPLEMENTARY MATERIAL

For supplementary material accompanying this paper visit http://doi.org/10.1017/S0950268815000965. click here to view supplementary material
