# Supplementary material for: Prevalent high-risk HPV infection and vaginal microbiota in Nigerian women
Source: Epidemiol Infect. 2015 Jun 11;144(1):123–37. doi: 10.1017/S0950268815000965 (PMC4659743; doi:10.1017/S0950268815000965)
Supplement: Supplementary file 1 [file S0950268815000965sup001.docx]

**SUPPLEMENTARY MATERIAL.**

**Table S1. Average abundance of phylotypes in each group**

| **Bacteria** | **Average Abundance in community (per cent)** | | | |
| --- | --- | --- | --- | --- |
|  | I | III | IV-B | VI |
| *Lactobacillus iners* | 8.90 | 64.25 | 4.01 | 0.13 |
| *Gardnerella vaginalis* | 1.15 | 6.89 | 20.68 | 11.48 |
| Proteobacteria | 9.70 | 8.00 | 5.36 | 77.51 |
| *Lactobacillus crispatus* | 66.48 | 0.82 | 2.30 | 0.02 |
| Pseudomonas | 4.85 | 3.69 | 5.83 | 0.03 |
| *Atopobium vaginae* | 1.81 | 1.32 | 6.51 | 0.20 |
| Gammaproteobacteria | 0.95 | 1.37 | 0.95 | 2.34 |
| *Leptotrichia amnionii* | 0.01 | 0.60 | 4.34 | 0.02 |
| *Megasphaera sp. type 1* | 0.01 | 1.25 | 2.74 | 0.03 |
| Prevotella genogroup 2 | 0.04 | 0.25 | 2.99 | 0.02 |
| *Prevotella bivia* | 0.08 | 0.28 | 2.80 | 0.33 |
| Prevotella genogroup 1 | 0.00 | 0.43 | 2.50 | 0.07 |
| Bifidobacteriaceae | 0.12 | 0.76 | 2.01 | 0.62 |
| Clostridiales | 0.11 | 0.05 | 0.57 | 0.03 |
| Streptococcus | 0.14 | 0.17 | 2.24 | 1.14 |
| *Lactobacillus jensenii* | 0.54 | 2.54 | 0.69 | 0.01 |
| BVAB1 | 0.00 | 0.08 | 2.14 | 0.15 |
| Bifidobacterium | 0.01 | 0.01 | 1.02 | 0.01 |
| *Escherichia coli* | 0.06 | 0.20 | 0.77 | 0.04 |
| BVAB2 | 0.00 | 0.08 | 1.93 | 0.03 |
| Eubacterium | 0.01 | 0.02 | 0.14 | 0.00 |
| Prevotella genogroup 3 | 0.00 | 0.17 | 1.81 | 0.00 |
| *Lactobacillus gasseri* | 0.09 | 0.04 | 1.91 | 0.00 |
| *Sneathia sanguinegens* | 0.00 | 0.19 | 1.72 | 0.06 |
| Enterobacteriaceae | 0.31 | 0.48 | 0.32 | 0.61 |
| Bacteria | 0.19 | 0.18 | 0.50 | 0.47 |
| *Veillonella montpellierensis* | 0.06 | 0.31 | 1.42 | 0.00 |
| Lactobacillus OTU1 | 1.11 | 1.06 | 0.51 | 0.04 |
| Actinobacteridae | 0.59 | 0.73 | 0.61 | 1.18 |
| *Aerococcus christensenii* | 0.01 | 0.32 | 1.00 | 0.00 |
| Veillonellaceae | 0.04 | 0.04 | 0.51 | 0.04 |
| Prevotella | 0.03 | 0.22 | 0.91 | 0.12 |
| Bacteroides | 0.00 | 0.01 | 0.03 | 0.01 |
| *Megasphaera sp. type 2* | 0.00 | 0.05 | 1.00 | 0.00 |
| *Streptococcus agalactiae* | 0.47 | 0.15 | 0.84 | 0.15 |
| *Dialister sp. type 2* | 0.00 | 0.09 | 0.84 | 0.01 |
| Prevotella genogroup 4 | 0.00 | 0.07 | 0.78 | 0.01 |
| Bacteroidales | 0.02 | 0.02 | 0.03 | 0.04 |
| Peptostreptococcus | 0.01 | 0.11 | 0.71 | 0.05 |
| *Streptococcus anginosus* | 0.02 | 0.06 | 0.69 | 0.82 |
| Eggerthella | 0.00 | 0.08 | 0.71 | 0.01 |
| *Parvimonas micra* | 0.00 | 0.06 | 0.70 | 0.00 |
| Actinomycetales | 0.27 | 0.23 | 0.41 | 0.50 |
| Gemella | 0.00 | 0.09 | 0.60 | 0.00 |
| *Anaerococcus tetradius* | 0.02 | 0.06 | 0.58 | 0.05 |
| Megasphaera | 0.01 | 0.14 | 0.51 | 0.01 |
| Porphyromonas | 0.01 | 0.05 | 0.41 | 0.04 |
| *Prevotella melaninogenica* | 0.00 | 0.06 | 0.56 | 0.00 |
| Staphylococcus | 0.14 | 0.07 | 0.48 | 0.02 |
| Coriobacteriaceae | 0.00 | 0.02 | 0.14 | 0.00 |
| Sneathia | 0.01 | 0.05 | 0.47 | 0.00 |
| Bacillales | 0.05 | 0.03 | 0.40 | 0.01 |
| Peptoniphilus | 0.01 | 0.03 | 0.40 | 0.05 |
| Mobiluncus | 0.00 | 0.00 | 0.36 | 0.02 |
| Dialister | 0.01 | 0.02 | 0.21 | 0.03 |
| Enterococcus | 0.02 | 0.12 | 0.21 | 0.02 |
| Veillonella | 0.02 | 0.05 | 0.26 | 0.04 |
| *Streptococcus salivarius* | 0.00 | 0.04 | 0.00 | 0.38 |
| *Lactobacillus vaginalis* | 0.79 | 0.24 | 0.07 | 0.00 |
| Dialister sp. type 1 | 0.01 | 0.06 | 0.27 | 0.01 |
| *Raoultella planticola* | 0.00 | 0.01 | 0.29 | 0.00 |
| Firmicutes | 0.02 | 0.03 | 0.06 | 0.01 |
| *Prevotella disiens* | 0.00 | 0.02 | 0.28 | 0.01 |
| Atopobium | 0.05 | 0.06 | 0.22 | 0.01 |
| *Peptoniphilus lacrimalis* | 0.00 | 0.01 | 0.25 | 0.01 |
| Pseudomonadales | 0.16 | 0.10 | 0.09 | 0.22 |
| *Finegoldia magna* | 0.06 | 0.11 | 0.16 | 0.20 |
| BVAB3 | 0.00 | 0.00 | 0.25 | 0.01 |
| *Bifidobacterium bifidum* | 0.00 | 0.00 | 0.01 | 0.03 |
| Ureaplasma | 0.04 | 0.15 | 0.10 | 0.02 |
| Clostridiales Family XI. Incertae Sedis | 0.01 | 0.03 | 0.12 | 0.05 |
| *Fusobacterium nucleatum* | 0.00 | 0.12 | 0.12 | 0.00 |
| *Mycoplasma hominis* | 0.00 | 0.10 | 0.11 | 0.00 |
| *Bacteroides uniformis* | 0.00 | 0.00 | 0.00 | 0.00 |
| Fusobacteriaceae | 0.01 | 0.03 | 0.13 | 0.01 |
| *Prevotella buccalis* | 0.00 | 0.01 | 0.14 | 0.02 |
| *Streptococcus anginosus group* | 0.00 | 0.01 | 0.12 | 0.15 |
| *Fusobacterium gonidiaformans* | 0.00 | 0.00 | 0.14 | 0.00 |
| Aerococcus | 0.01 | 0.05 | 0.10 | 0.00 |
| Bacilli | 0.01 | 0.01 | 0.02 | 0.01 |
| *Eubacterium rectale* | 0.00 | 0.00 | 0.01 | 0.00 |
| Actinomycetaceae | 0.01 | 0.01 | 0.09 | 0.00 |
| Lactobacillales | 0.02 | 0.03 | 0.04 | 0.01 |
| *Peptostreptococcus stomatis* | 0.00 | 0.01 | 0.08 | 0.04 |
| Arcanobacterium | 0.00 | 0.00 | 0.08 | 0.00 |
| Anaerococcus | 0.00 | 0.01 | 0.06 | 0.01 |
| Prevotella genogroup 6 | 0.00 | 0.00 | 0.06 | 0.00 |
| *Peptoniphilus asaccharolyticus* | 0.01 | 0.01 | 0.05 | 0.00 |
| Actinobacteria .class. | 0.02 | 0.02 | 0.02 | 0.03 |
| Prevotella genogroup 7 | 0.00 | 0.00 | 0.05 | 0.00 |
| *Anaerococcus vaginalis* | 0.02 | 0.02 | 0.03 | 0.02 |
| Pseudomonas putida group | 0.02 | 0.03 | 0.02 | 0.00 |
| *Porphyromonas sp. type 1* | 0.00 | 0.00 | 0.04 | 0.00 |
| Fusobacterium | 0.00 | 0.01 | 0.03 | 0.00 |
| Lachnospiraceae | 0.00 | 0.00 | 0.00 | 0.00 |
| *Eubacterium eligens* | 0.00 | 0.00 | 0.00 | 0.00 |
| *Eubacterium siraeum* | 0.00 | 0.00 | 0.00 | 0.00 |
| Actinomyces | 0.01 | 0.01 | 0.02 | 0.01 |
| *Lactobacillus coleohominis* | 0.06 | 0.02 | 0.01 | 0.00 |
| *Acinetobacter calcoaceticus.baumannii complex* | 0.01 | 0.01 | 0.01 | 0.00 |
| Mycoplasmataceae | 0.00 | 0.02 | 0.01 | 0.00 |
| Mycoplasma | 0.00 | 0.01 | 0.01 | 0.00 |
| *Varibaculum cambriense* | 0.00 | 0.00 | 0.02 | 0.01 |
| *Corynebacterium accolens* | 0.01 | 0.01 | 0.01 | 0.01 |
| Roseburia | 0.00 | 0.00 | 0.00 | 0.00 |
| *Streptococcus equinus* | 0.00 | 0.00 | 0.01 | 0.02 |
| *Veillonella parvula* | 0.00 | 0.00 | 0.01 | 0.00 |
| *Lactobacillus salivarius* | 0.00 | 0.00 | 0.01 | 0.00 |
| *Streptococcus mitis* | 0.00 | 0.00 | 0.01 | 0.00 |
| *Agrobacterium tumefaciens* | 0.00 | 0.00 | 0.00 | 0.01 |
| *Porphyromonas asaccharolytica* | 0.00 | 0.00 | 0.01 | 0.00 |
| *Lactobacillus helveticus* | 0.05 | 0.00 | 0.00 | 0.00 |
| candidate division TM7 | 0.00 | 0.00 | 0.01 | 0.00 |
| Sutterella | 0.00 | 0.00 | 0.00 | 0.00 |
| *Campylobacter ureolyticus* | 0.00 | 0.00 | 0.01 | 0.00 |
| *Atopobium minutum* | 0.00 | 0.00 | 0.01 | 0.00 |
| *Lactobacillus fermentum* | 0.00 | 0.00 | 0.00 | 0.05 |
| *Acinetobacter calcoaceticus* | 0.00 | 0.00 | 0.00 | 0.00 |
| *Lactobacillus mucosae* | 0.02 | 0.00 | 0.00 | 0.00 |
| *Bacteroides coagulans* | 0.00 | 0.00 | 0.01 | 0.00 |
| *Pseudomonas aeruginosa* | 0.00 | 0.00 | 0.00 | 0.00 |
| *Actinomyces meyeri* | 0.00 | 0.00 | 0.00 | 0.00 |
| *Porphyromonas bennonis* | 0.00 | 0.00 | 0.00 | 0.00 |
| *Catonella morbi* | 0.00 | 0.00 | 0.00 | 0.00 |
| *Sutterella wadsworthensis* | 0.00 | 0.00 | 0.00 | 0.00 |
| *Staphylococcus haemolyticus* | 0.00 | 0.00 | 0.00 | 0.00 |
| *Acinetobacter baumannii* | 0.01 | 0.00 | 0.00 | 0.00 |
| *Mobiluncus curtisii* | 0.00 | 0.00 | 0.00 | 0.00 |
| *Actinomyces neuii* | 0.00 | 0.00 | 0.00 | 0.00 |
| Lactobacillaceae | 0.00 | 0.00 | 0.00 | 0.00 |
| *Streptococcus intermedius* | 0.00 | 0.00 | 0.00 | 0.00 |
| *Streptococcus mutans* | 0.00 | 0.00 | 0.00 | 0.00 |
| *Weissella paramesenteroides* | 0.00 | 0.00 | 0.00 | 0.00 |
| *Atopobium rimae* | 0.00 | 0.00 | 0.00 | 0.00 |
| *Pediococcus acidilactici* | 0.00 | 0.00 | 0.00 | 0.00 |
| *Lactobacillus casei* | 0.00 | 0.00 | 0.00 | 0.00 |
| *Actinomyces urogenitalis* | 0.00 | 0.00 | 0.00 | 0.00 |
| Dialister sp. type 3 | 0.00 | 0.00 | 0.00 | 0.00 |
| *Aerococcus viridans* | 0.00 | 0.00 | 0.00 | 0.00 |
| Bacteroidetes.Chlorobi group | 0.00 | 0.00 | 0.00 | 0.00 |
| *Enterococcus faecium* | 0.00 | 0.00 | 0.00 | 0.00 |
| *Porphyromonas endodontalis* | 0.00 | 0.00 | 0.00 | 0.00 |
| *Staphylococcus epidermidis* | 0.00 | 0.00 | 0.00 | 0.00 |
| *Bifidobacterium breve* | 0.00 | 0.00 | 0.00 | 0.00 |
| *Staphylococcus capitis* | 0.00 | 0.00 | 0.00 | 0.00 |
| *Serratia marcescens* | 0.00 | 0.00 | 0.00 | 0.00 |
| *Alloscardovia omnicolens* | 0.00 | 0.00 | 0.00 | 0.00 |
| *Staphylococcus aureus* | 0.00 | 0.00 | 0.00 | 0.00 |
| *Veillonella atypica* | 0.00 | 0.00 | 0.00 | 0.00 |
| *Roseburia hominis* | 0.00 | 0.00 | 0.00 | 0.00 |
